# The current understanding of the immune landscape relative to radiotherapy across tumor types

**DOI:** 10.3389/fimmu.2023.1148692

**Published:** 2023-03-16

**Authors:** Chrysanthi Iliadi, Laurine Verset, Christelle Bouchart, Philippe Martinive, Dirk Van Gestel, Mohammad Krayem

**Affiliations:** ^1^ Department of Radiation Oncology, Institut Jules Bordet, Université Libre de Bruxelles (ULB), Hôpital Universitaire de Bruxelles (H.U.B), Brussels, Belgium; ^2^ Laboratory of Clinical and Experimental Oncology (LOCE), Institut Jules Bordet, Université Libre de Bruxelles (ULB), Hôpital Universitaire de Bruxelles (H.U.B), Brussels, Belgium; ^3^ Department of Pathology, Institut Jules Bordet, Université Libre de Bruxelles (ULB), Hôpital Universitaire de Bruxelles (H.U.B), Brussels, Belgium

**Keywords:** radiotherapy, cancer, systemic immune response, immune tumor microenvironment, immune landscape, biomarker, immune cells

## Abstract

Radiotherapy is part of the standard of care treatment for a great majority of cancer patients. As a result of radiation, both tumor cells and the environment around them are affected directly by radiation, which mainly primes but also might limit the immune response. Multiple immune factors play a role in cancer progression and response to radiotherapy, including the immune tumor microenvironment and systemic immunity referred to as the immune landscape. A heterogeneous tumor microenvironment and the varying patient characteristics complicate the dynamic relationship between radiotherapy and this immune landscape. In this review, we will present the current overview of the immunological landscape in relation to radiotherapy in order to provide insight and encourage research to further improve cancer treatment. An investigation into the impact of radiation therapy on the immune landscape showed in several cancers a common pattern of immunological responses after radiation. Radiation leads to an upsurge in infiltrating T lymphocytes and the expression of programmed death ligand 1 (PD-L1) which can hint at a benefit for the patient when combined with immunotherapy. In spite of this, lymphopenia in the tumor microenvironment of ‘cold’ tumors or caused by radiation is considered to be an important obstacle to the patient’s survival. In several cancers, a rise in the immunosuppressive populations is seen after radiation, mainly pro-tumoral M2 macrophages and myeloid-derived suppressor cells (MDSCs). As a final point, we will highlight how the radiation parameters themselves can influence the immune system and, therefore, be exploited to the advantage of the patient.

## Introduction

In recent years, sequencing of different tumors has revealed a vast heterogeneity across different cancer types but also between patients with the same diagnosis, highlighting the need for personalized medicine (1). The recent re-evaluation of the cancer hallmarks emphasized the essential role of the tumor microenvironment (TME) in tumor progression (2). The TME is a complex network of different cell populations and the interactions between them. The main cell neighborhoods of the TME are the tumor-, stromal-, vasculature- and immune cells. These cellular elements interact and create an evolving and dynamic environment that determines the response to different therapeutic regimens. Therefore, it is evident that an analysis of the multiple cell components in the TME can help design the most effective therapeutic strategy (3). The immune cells have a dual role in shaping the tumor by promoting or preventing its growth in a process named cancer immunoediting (4). In the framework of this process, the immune landscape, that is 1) the heterogeneous network of immune cells, 2) the immune components such as chemoattractants, and 3) other immunogenic factors such as tumor mutational burden (TMB), is widely studied (5). The balance of the different immune populations, the spatial localization, and the functional phenotype of the immune tumor microenvironment (iTME) establish the immune contexture (6). The immune landscape and contexture influence the response to treatment but are also contextually shaped by the therapeutic regimen itself.

Based on the immune landscape of solid tumors, several pan-cancer classifications were developed: the four consensus molecular subtypes - CMS (ie CMS1 - microsatellite instability immune, CMS2 - canonical, CMS3 - metabolic and CMS4 - mesenchymal) (7), the six immune transcriptomics subtypes – IS (ie wound healing, IFN-γ dominant, inflammatory, lymphocyte depleted, immunologically quiet and TGF-β dominant) (8) and most recently the four immune/fibrotic TME subtypes (ie IE/F – Immune Enriched/Fibrotic, IE, F, D-Desert) (9). As these strategies take into account the characteristics of the tumor microenvironment, they try to achieve more effective patient stratification than the traditional classification based on the histological characteristics of the tumor and the TNM staging system (10).

**Figure 1:**
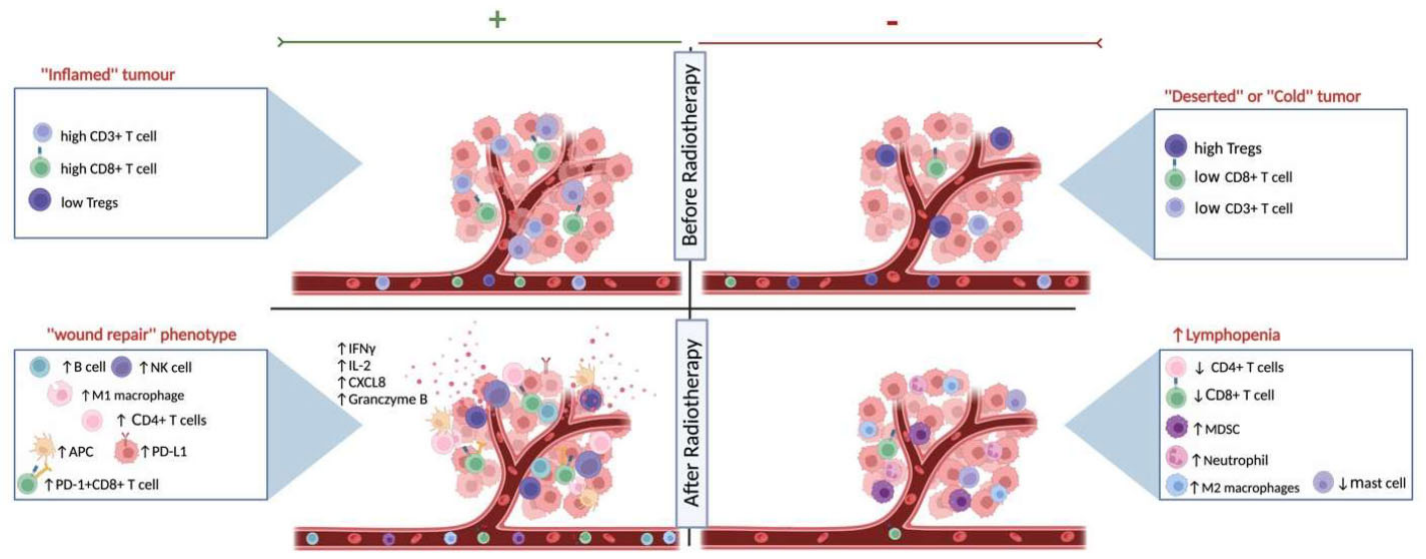
The effects of radiotherapy on the immune landscape. Schematic representation of the different states of systemic immune landscape and the tumor microenvironment before and after radiotherapy. As shown on the top left, the ‘‘inflamed immune’’ phenotype responds better to RT, as exhibited by higher immunoscores (CD3+ and CD8+ T cell densities), while the ‘‘deserted’’ or ‘‘cold’’ tumors on the top right respond less well to radiation therapy. The effects of RT on the immune landscape are influenced by the existing heterogeneous tumor microenvironment and the individual patient’s immune response. On the one hand, after RT, there might be a shift towards the wound healing signature (bottom left) and patients with this immune-hot phenotype have increased survival after treatment and might also be eligible for combination therapy with immunotherapy. Radiation can also cause a lymphopenic systemic and iTME landscape (bottom right), resulting in a worse prognosis. Created with BioRender.com.

Radiotherapy (RT) is one of the standard therapeutic regimens that take advantage of the damaging effect of ionizing radiation on DNA, leading to proliferative cell death and direct killing of the tumor cells (11, 12). Indirectly, RT leads to contrasting results shaping the iTME either to an immunogenic or to an immunosuppressive phenotype (see **Figure 1**). Shifting the delicate balance towards the immunogenic phenotype, radiation-related killing of tumor cells leads to the release of neoantigens and damage-associated molecular patterns (DAMPs). These signals, in turn, lead to an increase in antigen presentation and therefore activation of the innate immune system, an increase of CD8+ cytotoxic T cell infiltration, and inhibition of immunosuppressive cells (13). Contrariwise, tipping the scale towards the immunosuppressive phenotype, the use of radiation results most of the time in the direct killing of T lymphocytes inside the radiation field, increments the myeloid-derived suppressor cells (MDSCs) and regulatory T cells (Tregs) infiltration, and activates cancer-associated fibroblasts (CAFs) (14) through the TGF-β pathway, therefore, promoting tumor growth (15–17). Interestingly, RT has not only a modulating effect on the iTME but also systemically alters the immune profile of the patient. As was shown in a meta-analysis study across different cancer types, RT resulted in a systemic reduction of CD3+ and CD4+ peripheral T cells one month after the last treatment (18). Moreover, the role of radiation in the immune status of the TME can be exploited in the form of *in-situ vaccination* depending on the dose and fractionation schedule of radiation, the pre-existing immune profile of the tissue and patient, and the radiosensitivity of the tumor itself (19, 20). The *in-situ* vaccination can lead to systemic effects with a few examples of abscopal effect (21).

This review will examine the complexity of the systemic and local immune environment in different types of cancer. Our discussion will focus on how radiation shapes the immune landscape. In addition, we will unravel the potential prognostic and predictive insight we can gain from the iTME and systemic immune profile of the patient to guide therapeutic decisions (see **Table 1**).

**Table 1:** Immune landscape elements associated with prediction and prognosis in patients that underwent radiotherapy.

Cancer Type	Sample type	Number of patients	Treatment	Methods	Response assessment	Immune landscape parameter	Reference number
Esophageal Cancer	PBMCs	297	CRT	FC	Better OS	Low Treg High CD4+CD8+	(22)
	Serum	63	RT	ELISA	Response *VS* Non-Response to treatment	Elevated levels of IL-2 and IFN-γ	(23)
	FFPE samples	81	CRT	IHC	Response to nCRT	High density of CD8+ T cells Foxp3+ T cells and PD-1+ T cells	(24)
	FFPE samples	31	CRT	IHC and qRT-PCR	–	Increase in CD8+ T cells after CRT	(25)
	FFPE samples	123	CRT	IHC	Worse OS	High CD8+ T cell inflitration	(26)
nasopharyngeal carcinoma	Tissue samples and peripheral blood	36	CRT	TCRβ sequencing	Longer DFS	Higher number of mucosa-resident ITCs	(27)
limited-stage small cell lung cancer (LS-SCLC)	PBMCs	98	CRT	FC	Higher PFS	Higher number of CD4+CD45RA+, CD8+CD38+	(28)
stage I NSCLC	PBMCs	19	SBRT	TCR sequencing	Poor MFS	Lower TCR diversity	(29)
Lung adenocarcinoma (LUAD)	mRNA data	423	RT	Functional genomics – differential expression analysis	Better response to RT	Low risk tumor-infiltrating B lymphocyte-specific genes (TILBSig)	(30)
hepatocellular carcinoma	Blood sample	164	SIRT	Blood count	Better OS	NLR < 7.2	(31)
locally advanced rectal cancer (LARC)	Biopsies	249	CRT	IHC	High DFS	High Immunoscore (CD3+ and CD8+ T cells in the tumor core and invasive margin)	(32)
Rectal Cancer	Biopsies and resection specimens	53	CRT	Functional genomics: gene expression profiling	Response to RT/CRT	‘‘hot’’ iTME phenotype after treatment	(33)
rectal adenocarcinoma	FFPE recession samples, PBMCs or histologically normal rectal tissue	17	CRT	IHC	Response to CRT	Higher infiltration in CD8+ T cells	(34)
8 cancer types	Published literate	>10.328	Various	Meta-analysis	Poor OS	Low Immunoscore	(35)
Rectal Cancer	FFPE	166	CRT	IHC	Better DFS and OS	High Immunoscore (densities of CD3+ and CD8+ lymphocytes)	(36)
Pancreatic Cancer	PBMCs	66	CRT	ELISA or flow cytometry–based multiplex bead arrays	Greater mOS	above-median CXCL8 serum levels (>29.8 pg/ml) and	(37)
					Prolonged mOS	Above-median pretreatment NK cell numbers (NKhigh: CIBERSORT fraction, >4.5%)	
Pancreatic Cancer	FFPE tissue blocks	70	nCRT	mIHC/IF	longer RFS	low density of M2-type TAMs	(38)

RT, radiotherapy; CRT, chemoradiotherapy; SBRT, stereotactic body radiotherapy; SIRT, Selective internal radiation therapy; nCRT, NeoAdjuvant Therapy; OS, overall survival; mOS, median overall survival; PFS, progression free survival; DFS, disease free survival; FFPE, Formalin Fixated Paraffin Embedded Sample; PBMCs, Peripheral Blood Mononuclear Cells; mRNA, messenger ribonucleic acid; ELISA, Enzyme-Linked Immunosorbent Assay; IHC, Immunohistochemistry; FC, Flow Cytometry; MFS, Metastasis Free Survival; NLR, Neutrophil-to-Lymphocyte Ratio; mIHC/IF, Multiplex Immunohistochemistry/Immunofluorescence. “-” There is no correlation observed between the alteration in iTME and the parameters for evaluating response in patients.

## Methods

The Pubmed database was searched for literature articles and reviews published between January 1^st^, 2012 and October 31^st^, 2022 that investigate the effect of radiation therapy on the immune landscape of various malignancies. The search strategy included vocabulary related to radiotherapy (e.g., radiation, chemoradiation, fractionation) and to the immune system (e.g., immune cell, innate and adaptive immunity, infiltration, cytotoxicity). Additionally, each type of cancer was used as a keyword accompanied by the aforementioned terms to search for related information. Moreover, reference lists of selected reviews were screened to be redirected to the original study.

Results describing either prospective or retrospective settings were evaluated in full-text articles. Throughout this review, we make a distinction between the systemic immune landscape that includes changes in the peripheral blood mononuclear cells (PBMCs) and the immune landscape of the tumor microenvironment for which mainly formalin-fixed paraffin-embedded (FFPE) or fresh samples of the tumors are evaluated. The methods most commonly used for the analysis of the immune landscape are flow cytometry, immunohistochemistry or tissue microarrays, and transcriptomics analysis. To emphasize the importance of further investigation in pre-clinical models, we separated the data produced by such studies from data on human samples when available. Moreover, all treatment regimens were included, RT alone as well as CRT without restrictions on cytotoxic agents used. Finally, we included data describing the difference between treated and untreated specimens or samples before treatment (biopsies).

Finally, the records of the database ClinicalTrials.gov of the U.S. National Library of Medicine were searched to identify clinical trials that involved a combination of radiotherapy and immune checkpoint inhibitors for the following types of cancer. The search focused specifically on double-arm trials, which involve concurrent radiotherapy and immunotherapy in at least one experimental arm, as these trials can provide a more rigorous evaluation of treatment efficacy. By including a control group, the double-arm trial can help to establish whether any observed benefits are due to the combination of treatments, or whether they would have occurred with one of the treatments alone. Clinical trials classified as ‘withdrawn’ or ‘unknown’ were excluded. The results are presented in **Table 2**.

**Table 2:** Clinical trials that strive to show therapeutic benefit of combining immunotherapy and radiotherapy.

Cancer Type	Immune Target	Antibody	RT Regimen	Experimental Arms	Primary Endpoint	Therapeutic Benefit or Clinical Trial Status *	Identifier or Reference
Head and Neck Cancer	PD-L1	Avelumab	69.96 Gy in 2.12 Gy/day over 6.5W	ICI + RT -cetuximab *VS* SOC	PFS	Active, not recruiting	NCT02999087
	PD-1 and CTLA-4	Nivolumab and Ipilimumab	56-66 Gy	Neoadjuvant/Adjuvant ICI+CRT *VS* Surgical resection + Adjuvant CRT	DFS	Active, not recruiting	NCT03700905
Lung Cancer	PD-L1	Durvalumab	at least 60 Gy	CRT+ICI *VS* CRT+placebo	PFS	Median PFS was 16.8 months with ICI *VS* 5.6 months with placebo	NCT02125461 (39)
	PD-1	Pembrolizumab	50 Gy	CRT *VS* ICI and dose-painted radiotherapy	PFS	Recruiting	NCT03523702
	PD-L1	Atezolizumab	Radiotherapy up to 21 days	ICIs + RT *VS* ICIs	ORR	Recruiting	NCT03337698
	PD-1	Pembrolizumab	18 Gy in 3 X 6 Gy	ICI+ CRT *VS* ICI+ CT	OS	Recruiting	NCT03774732
	PD-L1	Atezolizumab	61.2 GY	ICI + CRT + surgery *VS* CRT + surgery	pCR	Recruiting	NCT04989283
	PD-L1	Atezolizumab	3D-CRT or IMRT BID for 3W	CRT+IT *VS* CRT	OS	Recruiting	NCT03811002
	PD-L1	Durvalumab	15 Gy in 10 fractions	ICI with low-dose PCI *VS* ICI	Reduction of incidence of brain metastases	Recruiting	NCT04597671
	PD-1	Pembrolizumab	3 x 8 Gy	RT + ICI *VS* ICI	ORR	The ORR at 12 weeks was 18% in the control arm *vs* 36% in the experimental arm (P = .07)	(40) NCT02492568
Lung Cancer and Melanoma	PD-1 and CTLA-4	Nivolumab and Ipilimumab	1 x 18-22 Gy (18-22 Gy) or 5 x 6 Gy (30 Gy).	Stereotactic radiosurgery and ICI *VS* ICI	CNS-specific PFS	Recruiting	NCT05522660
Lung Cancer and Colorectal Cancer	PD-L1 and CTLA-4	Durvalumab and Tremelimumab	Not Specified	ICIs *VS* ICIs + big dose RT *VS* ICIs + low dose RT	ORR	Active, not recruiting	NCT02888743
Esophageal Cancer	PD-1/PD-L1	Not specified	50-66G/1.8-2.2Gy/25-30f	CRT *VS* CRT + ICI	OS in 1Y, 2Y, 3Y and 5Y	Recruiting	NCT04821778
	PD-1	Camrelizumab	50Gy/30f	ICI + CRT *VS* ICI + CT	ORR	Not yet recruiting	NCT05624099
	PD-1	Camrelizumab	50-50.4G, 1.8-2 Gy, 5d/w	ICI + CRT *VS* Placebo + CRT	PFS	Not yet recruiting	NCT04404491
	PD-L1	Durvalumab	50 Gy	CRT + ICI *VS* CRT	cPFS	Recruiting	NCT03777813
	PD-1	Camrelizumab	8 Gy/time, 3 -5 times	CRT + IT *VS* CT+IT	PFS	Not yet recruiting	NCT05183958
Cervical Cancer	PD-1	Serplulimab	80 Gy for small-volume tumors or 85 Gy for larger-volume tumors	IT + CRT *VS* CRT	PFS	Not yet recruiting	NCT05173272
Breast Cancer	PD-1	Pembrolizumab	Low-Dose or High-Dose RT	ICI *VS* ICI + Low-Dose RT *VS* ICI + High-Dose RT	TILs and pCR-LN	Recruiting	NCT04443348
	PD-1	Pembrolizumab	Focal hypo-fractionated RT 8 Gy x 3 fractions	RT *VS* RT+ICI *VS* RT+Ftl-3 ligand *VS* RT+Ftl-3+ ICI	Tolerability and pCR/cCR	Recruiting	NCT03804944
Ovarian Cancer	CD-40 and PD-L2	APX005M and Carboplatin	0.5 Gy/fr; days 1 and 15 q4 wks x 6 cycles. Maximum 24 wks of therapy; total dose 6 Gy.	SOC *VS* APX005M *VS* APX005M+RT	ORR	Not yet recruiting	NCT05201001
Prostate Cancer	PD-1 and CTLA-4	Nivolumab and Ipilimumab	8 Gray (Gy) x 3	RT + ICIs *VS* ICIs	ORR and PSA RR	Recruiting	NCT05655715
	PD-L1	Durvalumab	SBRT will be started one month after ICI in 3fr	RT + ICI *VS* RT	2-year PFS	Recruiting	NCT03795207
Pancreatic Cancer	PD-1	Pembrolizumab	50.4 Gy in 28fr over 28 days	nCRT+ICI *VS* nCRT	Number of TILS per hpf and DLT	Recruiting	NCT02305186
	PD-L1 (and TGF-β)	Bintrafusp alfa (M7824)	RT will be starting on Day 17 (+5 days)	ICIs + RT *VS* ICIs	DLT and Recommended Phase 2 Dose and BOR	Terminated (Study closed to accrual due to the worsening risk: benefit ratio for participants receiving bintrafusp alfa (M7824)	NCT04327986
Hepatocellular Carcinoma	PD-1	Sintilimab	30-54 Gy in 3-6fr over 1-2W	RT+ICI *VS* RT	24-week PFS	Recruiting	NCT04167293
Melanoma, Non-Hodgkin Lymphoma, Colorectal Cancer	CTLA-4	Ipilimumab	10 Gy x 3 fractions	ICI + RT *VS* ICI	DLT	0% Serious AE - Terminated (Planned Future Study)	NCT01769222
Advanced Malignancies	CD-137 (4-1BB), PD-L1 and CD137 (OX40)	Utomilumab, Avelumab, Ivuxolimab	Patients undergo RT on days -5 to -1/Dose Not Specified	ICIs *VS* ICIs+RT	Incidence of adverse events and Evaluation of CD8 immune biomarkers assessed in tumor and blood	Active, not recruiting	NCT03217747

RT, radiotherapy; CT, chemotherapy; CRT, chemoradiotherapy; SOC, Standard of Care; ICI, Immune Checkpoint Inhibitor; OS, overall survival; PFS, progression free survival; pCR-LN, Pathological Complete Response in the Lymph Nodes; ORR, Overall Response Rate; BOR, Best Overall Response; RR, Response Rate; DLT, Dose Limiting Toxicities; AE, Adverse Effects; TILS, Tumor Infiltrating Lymphocytes; PSA, Prostate-Specific Antigen; HPF, High Powered Field.

## Immune landscape and radiotherapy

### Parameters of the radiotherapy regimen that can influence the immune landscape

It is estimated that more than half of all cancer patients receive radiation therapy (11, 41). In a radiotherapy regimen, a variety of parameters can vary, such as the moment of treatment (neoadjuvant/preoperative or adjuvant treatment), the combination with other treatments like chemotherapy and/or immunotherapy, and the fractionation schedule, which altogether can result in a whole range of total doses at the end of treatment and treatment lengths.

As part of the modulation of the immune signature, fractionation plays an important role. A single dose of RT, compared to a multifractionated schedule, promotes a more immunogenic phenotype in prostate cancer cells *in vitro* (42). Moreover, in a murine orthotopic model of pancreatic cancer, RT recruited a greater number of T cells than fractionated RT (43). In particular, RT given as a single dose of 25Gy resulted in higher infiltration of cytotoxic T cells (CD8+) compared to the four times 10 Gy per fraction regimen. It is most interesting to note that there was no difference in tumor growth between the two groups, suggesting that different RT schedules had no effect on T-cell activity but only on infiltration.

Furthermore, changes in the immune landscape following fractionated RT can be affected by age and target volumes, especially when immune-related volumes are involved, such as blood vessels and bone marrow (44). As aging affects immune cells (25), it is only natural that the treatment’s impact on the immune landscape will also be influenced by aging. In a small cohort of prostate cancer patients treated with RT was studied to determine if the changes in immune cell subsets were influenced by age. CD4+ effector cells and patient age exhibited a moderately positive correlation, but not in the exploratory cohort (44). So far no strong relationship between age and radiation effects on immunity has been demonstrated. More coherent results derive from the relationship between target volume and immune landscape after RT. Lymphopenia is a common side-effect of RT and smaller target volumes are likely to affect less the immune landscape than larger ones (45).

Treatment planning variables such as the interval between RT and surgery and of course the addition of chemotherapy can also influence the iTME. These parameters were explored in rectal cancer (46), where less cytotoxic T cells and T helper cells infiltrated tumors following a shorter radiotherapy-to-surgery interval compared to the longer waiting time after an equal radiation dose. In addition, in the same study, CRT led to a significant decrease in T regulatory cell infiltration, demonstrating the combination to work synergistically to reverse immune suppression.

It can be challenging to draw conclusions about the state of the tumor’s immune status after radiotherapy, especially when we rely on biopsies, which are often not representative of the whole tumor mass. Moreover, the higher density of CD4+ and CD8+ T cells in surgical samples was correlated with the use of postoperative radiotherapy and good prognosis in a cohort of PDAC patients when large-section histopathology (LHS) slides were compared to small-section histopathology (SSH) slides that enclose less information on the TME. Therefore, the area that is covered during the analysis of immune staining can also be of great significance when it comes to evaluating prediction and/or progression.

It is crucial to thoroughly investigate the radiotherapy parameters that influence the iTME and systemic immune landscape, especially when combining radiation with immunotherapy for therapeutic purposes. For instance, a study involving lung cancer patients administered SBRT (3 doses of 8 Gy) in combination with pembrolizumab (anti-PD-1) demonstrated a doubling of the overall response rate (ORR) (40). Ongoing clinical trials are currently exploring the therapeutic benefits of this combination (see **Table 2**). A better understanding of how radiotherapy regimen parameters affect the synergy with immunotherapy could provide insights into the success or failure of such clinical trials (47).

### Esophageal cancer

Esophageal (EC) and esophagogastric junction (EGJ) cancers are tumors that develop along the esophagus with the most common types being squamous cell carcinoma (SCC) and adenocarcinoma (ADC) respectively. Their standard-of-care treatment for early-stage EC is endoscopic resection or surgical resection. Neoadjuvant chemoradiotherapy (nCRT) and surgery are recommended for more advanced stages (48) while immunotherapy with pembrolizumab can be as concurrent treatment as well. The results for immunotherapy are mixed as the anti-PD-1 reagent did not confer clinical benefit to patients with advanced PD-L1-positive gastroesophageal cancer (49). Therefore, much of the research in oesophageal cancer has been focused on understanding the immune landscape of the tumor in order to better stratify patients for immune checkpoint inhibitors (ICI) (50, 51).

#### Systemic immune landscape of EC and radiotherapy

A retrospective study using data from PBMCs of patients with non-operative EC that underwent chemoradiotherapy (CRT) showed that low density in Tregs and high concentration of double-positive (DP) CD4+ CD8+ T cells to be two independent predictive factors for response to CRT (22). Interestingly, the DP T cells represent a rarely studied subpopulation of immune cells that are found in cancer patients’ blood and their role is not entirely clear as to whether they are immunosuppressive or cytotoxic (52). In metastatic colorectal cancer, this subpopulation has been found to favor immunosuppression and tumor growth (53) while in urological cancer it is correlated with differentiation of CD4+ naïve T cells to the pro-tumoral Th2 phenotype (54). In EC patients, high densities of DP T cells are associated with better outcomes, but further investigation is needed to uncover their precise role. Although Fei Lan et. all. Systemically observed a change after CRT, another group (55) observed no change in the density of CD4+, CD3+ and CD8+ T cells after only RT compared to only the chemotherapy group. These results might hint that the ablative effect on the PBMCs may be mainly attributed to the CRT combination and not to RT alone.

RT-induced immune reactions in patients with EC can be correlated with serum levels of two immunostimulatory cytokines, IL-2 and IFN-γ. These cytokines were found to be elevated during RT in responders compared to non-responders, in terms of better local control. Follow-up of these changes in PBMC immune populations or even better, in immunohistochemical profiles of primary tumors would be interesting (23).

Knowing that a radiation treatment results in severe lymphopenia in most EC patients, one always needs to consider that this systemic effect can also dampen the immune response in the tumor as well. Moreover, a high estimated dose of radiation to immune cells (EDRIC) was correlated with higher-grade lymphopenia also resulting in worse OS and PFS (56).

Another finding is the prognostic value of platelet to albumin ratio (PAR) which has been associated with worse overall survival (OS) and progression-free survival (PFS) among patients treated with RT for ESCC (57).

#### iTME of EC and radiotherapy

The dynamic changes that RT might impose on the tumor-infiltrating lymphocytes (TILs) have been explored retrospectively in FFPE samples of patients with EC treated with nCRT (24). In this study, Soeratram et al. classified the iTMEs according to the combined mean density of cytotoxic T cells (CD8+), T regulatory cells (FOXP3+), and immune-checkpoint molecule PD-1 positive cells into: ‘‘inflamed’’ (or ‘‘hot’’), with most immune cells found in the tumor core (TC); ‘‘invasive margin’’ (or ‘‘excluded’’), when most tumor-associated immune cells (TAICs) were found on the invasive margin (IM); and ‘‘desert’’ (or ‘‘cold’’), consisting of samples with very low density in both compartments. In more than half of the nCRT recession samples (56%) the ‘‘inflamed’’ iTME was present, indicating a positive correlation between CRT and immune cell infiltration. Moreover, the most interesting studies are the ones that compare the pre-existing immune landscape on biopsies with the landscape after treatment in order to reveal dynamic changes. As such, based on pre-treatment biopsy and post-nCRT resection specimen pairs, an increase in the influx of CD8+ T cells was observed in the tumor epithelium, a finding which may aid treatment planning (24). This increase has also been found in a study by Kelly et al (25), when retrospectively comparing normal and malignant FFPE oesophageal epithelium post-nCRT samples with matched pre-treatment biopsies. The increase in lymphocyte infiltration like cytotoxic T cells and NK cells after CRT is commonly found in multiple studies on EC (58). It is important to further investigate the phenotype of these lymphocytes since on certain occasions there is an upregulation of immune checkpoint molecules as shown in ESCC patients treated with CRT where the increase in immune infiltration was parallel with an increase in one such molecule, the tyrosine-based inhibitory motif domain (TIGIT) (58). As for the two immune checkpoint therapeutic targets currently used in the clinic (59), there was an upregulation in programmed death-ligand 1 (PD-L1) expression after nCRT, yet the same trend was not evident for cytotoxic T-lymphocyte-associated protein 4 (CTLA-4). Based on complementary RNA data, nCRT increased IFNγ expression in tumor cells, perhaps as a result of the increased influx of cytotoxic T lymphocytes to balance the immune response by inducing tumor cells to express checkpoint molecules like PD-L1 and Indoleamine 2,3-Dioxygenase (IDO) (60). PD-L1 high expression on tumor cells was an independent prognostic factor for increased OS after RT (61). In another study (26) patients with esophageal adenocarcinoma (OAC) were divided based on their response to nCRT into poor, moderate, and good responders. Immunohistochemistry analysis was performed to quantify the expression of the following markers and subpopulations: CD3 (pan-T cell), CD4 (T helper cell), CD8 (62), FoxP3, and PD-L1 (expressed on cancer cells and antigen-presenting cells) (63, 64). Here, the tumor immune infiltrates, as seen by the CD3+ and CD8+ cells, were highly correlated with the cancer cell density. Surprisingly, only when the poor responders also displayed high levels of CD8+ T cells they had a poor OS, a trend that was not observed in the good/moderate responders. This result is contradictory to the consensus that high infiltration of cytotoxic T cells is favorable for the patient. A closer look at the phenotypic characteristics of these CD8+ T cells can shed more light on their specific function and on why their existence was negatively associated with OS.

In addition, the immune landscape can also be correlated with other aspects of the tumor microenvironment, and their relationship with RT can be analyzed collectively. One of those characteristics is the tumor mutational burden (TMB) which refers to the total number of mutations in a tumor, is proportional to neoantigen production, and is used as a marker of immunogenicity (65). Researchers categorized EC patients into high TMB (TMB-H) and low TMB (TMB-L) based on the extent of TMB in their tumors (66). The patients were then further separated based on whether they got RT was their number of Tregs was quantified. Those not receiving RT had an increased influx of Tregs in the TMB-H group compared to the TMB-L group. However, RT patients from both groups showed no difference in immunosuppressive cell infiltration, which may indicate a balancing effect of the RT (66).

Higher TILs have been suggested by multiple studies to be a reliable prognostic factor for the progression of the disease. In ESCC patients treated with CRT, a higher TILs density in the stroma of biopsy samples during therapy was correlated with increased 5-year disease-free survival (DFS). These results might be used to predict the response of the patients to CRT avoiding unnecessary surgery. A more detailed investigation of the heterogeneous TIL compartment clarifying the different subpopulations would enhance the predictive validity of this model (67).

In conclusion, data from EC shows radiation to possibly have a positive effect on the recruitment of immune cells in the tumor and more specifically on CD8+ T cell infiltration. In addition, RT-dependent upregulation of immune checkpoint molecules such as PD-L1 is a promising indication for the combination of RT with immunotherapy.

### Head and neck cancer

The term “head and neck cancer” refers to a group of cancers that are anatomically developed in the mouth, larynx, nose, sinuses, and throat, with squamous cell carcinoma (HNSCC) arising from epithelial cells lining the mucous membranes (68). In the case of HNSCC, radiation therapy is a standard treatment method that shows a better response in human papillomavirus (HPV) - positive patients than in HPV-negative ones (69). The virus-induced cancers display different biological and clinical characteristics but also dissimilar immune landscapes compared to their negative counterparts (70). A multicentre study analyzed the prognostic value of CD8+ and CD3+ T cells in correlation with HPV status in patients with HNSCC that underwent CRT treatment (71). This study concluded that the high density of cytotoxic T cells and HPV positivity are independent favorable factors for OS after CRT (71). This distinction of the virus status of HNSCC patients is therefore important to better exploit the unique characteristics of both subgroups and increase radiosensitivity.

#### Systemic immune landscape of HNSCC and radiotherapy

The combination of platinum-based chemotherapy and intensity-modulated radiotherapy (IMRT) in HPV-related oropharyngeal cancer shifted the balance into a systemic immunosuppressive state (72). After CRT an increased number of myeloid-derived suppressor cells (MDSC) and decreased numbers of CD8+ and CD4+ T cells were found while in the latter there was an upregulation in the expression of PD-1 immune-checkpoint molecule (72). These results partly come in agreement with the study of Sridharan et al. (73), which also showed an increase in circulating MDSC and in PD-1+ T cells. However, the systemic cytokine levels of CXCL10 and 16 respectively decreased and increased respectively in patients treated with radiation. CXCL10 is a cytokine believed to promote immunosuppression and tumor stemness (74, 75) while CXCL16 has a beneficial effect on cancer control since it promotes lymphocyte infiltration in the tumor (76). It is therefore clear that while an increase in immunosuppressive cell populations is observed, the cytokine profile changed to promote tumor regression.

J. Schuler and colleagues (77) compared the effect of CRT on the number of conventional CD4+ T cells (Tconv) and the subpopulation CD4+CD39+ Tregs that exercise their immunosuppressive action through the adenosine pathway (78). After CRT, the absolute number of CD4+ T cells and their percentage decreased, but within this Tconv population, CD4+CD39+ Treg cells increased, indicating that these cells were relatively resistant to the CRT regimen used. Complementary to this, they found upregulation in CD39 expression, an ectonucleotidase involved in the adenosine pathway, which led to the conclusion that CRT might stimulate the suppressor functions of Tregs. The persistence of a highly immunosuppressive population of Tregs after CRT was observed at different time points which might explain the frequent recurrence of HNSCC (77).

#### iTME of HNSCC and radiotherapy in human samples

For nasopharyngeal carcinoma (NPC) the standard of care therapy is concurrent chemoradiotherapy (CCRT) which can have immunostimulatory effects and increase the patient’s chances of survival (79). Chung et. al, focused on the Epstein-Barr viruspositive NPC, the most representative type of this cancer, and looked closely at the dynamic changes of the intratumorally T-cell clonotypes (ITCs) in search for predictive biomarkers for CCRT. They concluded that chemotherapy and RT combination drive the selection of ITCs to a remodeling of the unique TCRβ clonotypes. Surprisingly, CCRT does not lead to the expansion of the EBV-associated ITCs for an *in situ* vaccination effect as is widely believed to be the case (20, 27).

Most of the data available on head and neck cancer after RT are describing the systemic effect on the immune populations. This can always be seen as an indicator of the populations in the tumor contexture although we should be cautious when attempting to describe the iTME in head and neck cancer. Nevertheless, we can conclude that RT is increasing the number of immunosuppressive cell populations systemically like MSDCs and Tregs while also at the same time having a deleterious effect on CD8+ T cells. Intratumorally, an expansion in ITCs is observed therefore it would be very interesting to explore this further in relation to the systemic profile.

#### iTME of HNSCC and radiotherapy in preclinical models

As seen in patients, cells of the myeloid lineage show an expansion after RT (72). This has also been observed in a HNSCC murine model, where the density of CD11b+ infiltrated myeloid cells in the tumors increased after irradiation (80). Interestingly, when neutralizing antibodies against those bone marrow-derived infiltrates were used the tumors showed increased radiosensitivity and response to irradiation *in vivo* (81).

The interaction between the immune landscape and radiation in preclinical models of head and neck cancer is scarily known, but these studies remain important because they can provide insight into the driving pathways and sequence of events that shape the iTME.

### Lung cancer

According to GLOBOCAN estimates for 2020 lung cancer is the primary cause of cancer-related mortality (82). From traditional approaches like surgery and RT to immunotherapy with ICIs, there are many ways to combat lung cancer and lung metastases. For all patients with advanced surgically treatable lung cancer, CRT is part of the treatment regimen and has been proven successful in controlling the disease (83). Moreover, immunotherapy has been shown to be helpful in treating non-small cell lung cancer patients who are PD-L1 positive (84). It is imperative that patients are divided into distinct groups in order to determine the most appropriate treatment approach for each, and the focus is once again on the tumor microenvironment and immune system (85).

#### Systemic immune landscape of lung cancer and radiotherapy

In a cohort of limited-stage small cell lung cancer patients (LS-SCLC), CRT resulted in an increase of all T cells (CD3+), cytotoxic T cells (CD3+CD8+), activated T effector cells (CD8+CD38+) and NKT cells (28). The same retrospective study also showed a reduction in the percentage of T helper cells (CD3+CD4+), naïve T cells (CD4+CD45RA+), B cells, NK cells, and T helper/T effector cell ratio in the patients. Of all these subpopulations of immune cells, the high densities of naïve T cells and activated effector T cell 3 months post-CRT were both independent predictor factors of good progression-free survival (PFS). Moreover, in line with results from the peripheral blood of Epstein-Barr virus-associated nasopharyngeal cancer patients referred to above (27), Wu et al. (29), also examined the systemic effect of irradiation on the T-cell receptor (TCR) repertoire in stage I non-small-cell lung cancer (NSCLC) and concluded that after RT the number of unique TCR clones was decreased. More interestingly, the higher the diversity of the TCR clones at baseline the more likely it was for the patient to respond well to stereotactic body radiation therapy (SBRT).

When examining lung cancer cases it is important to mention the transient or prolonged lymphopenia frequently observed after radiation and which depends on the thoracic volume that is targeted (86) and on the fractionation regimen (87). Lymphopenia can be used as a prognostic factor for disease progression (87) but also as a predictive marker since the neutrophil-to-lymphocyte ratio is negatively correlated with response to immunotherapy (88) and can help stratify the patients for alternative treatments after radiation.

#### iTME of lung cancer and radiotherapy in human samples

To study the dynamic changes in the immune contexture, Zhou et al. (89) analyzed paired tumor samples from NSCLC before and after SBRT. RT improved the TCR repertoire diversity, but also increased the PD-L1 expression in the TME. Moreover, there was an augmentation in the expression of immune-regulating factors such as C-X-C motif chemokines (CXCL10 and CXCL16), interferons (IFN I and II) and interferon receptors (IFN IR and IFN IIR) intratumorally. Drifting away from the TCR clonotypes and collectively looking at the TIL populations Shirasawa et al. (90), retrospectively accessed the impact of RT on the PD-L1 expression and CD8+ T cell infiltration in NSCLC patients. PD-L1 expression in cancer cells did not show a particular trend, however, the density of CD8+ T cells increased after CRT, which can be exploitable in the scope of ICI therapy. Of note, a meta-analysis for the gene signature was performed in patients with lung adenocarcinoma (LUAD) who were divided into groups: RR (radiotherapy resistant)-patients showing poor response to radiotherapy and RS (radiotherapy sensitive)-patients presenting with better prognosis after therapy (30). T cells, monocytic lineages, B lineages, fibroblasts, cytotoxic lymphocytes, CD8+ T cells, endothelial cells, and NK cells were enriched in the RS group, while neutrophils were enriched in the RR patients.

#### iTME of lung cancer and radiotherapy in pre-clinical models

Preclinical studies about the impact of radiotherapy on the immune landscape in lung cancer are more prevalent than clinical studies on human lung cancer. Zhang et al. (91) assessed the effect of irradiation on the immune contexture in a syngeneic murine model of Lewis Lung carcinoma. They graded the infiltrated MDSCs and cytotoxic CD8+ T cells in the tumors to find an increased recruitment of MDSCs-mediated immunosuppression. To further access the causal link between MSDS and cytotoxic T-cell infiltration, they depleted the polymorphonuclear (PMN) – MDSCs or inhibited the expression of arginase 1 (ARG1) on these cells. Both these actions led to a flux of CD8+ T cells inside the tumors. They concluded that PMN-MDSCs are upregulated after irradiation that they suppress the immune cells of the TME in an arginase-related manner. The systemic effect of irradiation was further examined in a primary lung tumor mouse model in which the B cell density increased while CD8+ cytotoxic T cells decreased showing a direct effect of irradiation on innate immunity (92). In a similar manner, the effects of low-dose fractionated RT on the iTME were studied in an orthotopic murine model (93). As seen before, radiation induced an expansion in the number of MDSCs, neutrophils and F4/80+ macrophages and more specifically the MHC-II_hi_ anti-tumoral M1 subpopulation, while on the other hand, it significantly reduced the absolute number of CD8+ T cells in the spleen and lung. Further examination of the T cell compartment revealed expansion of Tregs (CS25+/CD127-) and PD-1+ T cells, suggesting a phenotypic shift towards immunosuppression that can be exploited for ICI therapy. Intratumorally, radiation recruits neutrophils as shown in a Lewis lung cancer adenocarcinoma murine model. The recruitment of tumor-associated neutrophils (TANs) leads to cytokine release and the subsequent CD8+ T cell infiltration. The increase in CD8+ T cell numbers in the TME is contradictory to the general trend of its systemic decrease after irradiation. This is a nice example of how a systemic effect does not necessarily translate in a similar manner intratumorally (94). To study the mechanism by which irradiation is affecting the immune cells in the tumor microenvironment, Wang et al. (95), performed *in vitro* experiments with NSCLC cell lines and CD8+ T cells from healthy donors. Contradictory to *in vivo* results that show an increase in PD-L1 expression in cancer cells, here they concluded that irradiation (IR) is augmenting CD8+ T cells immunity by suppressing PD-L1 expression in an IFNγ related manner.

Immunotherapy is frequently used in lung cancer, both alone and in combination with RT. Therefore, unraveling the immune landscape of the tumor could allow many patients to escape the adverse effects of the treatment. After radiation, there is an increase in the influx of MSDCs in the tumor but a balancing augmentation in the CD8+ T cells which comes in contradiction with the lymphopenia that is seen systemically.

### Breast cancer

Breast cancer (BC), the most common cancer in women, is treated based on tumor staging, size, location, and the patient’s health and preferences. Early-stage BC is usually treated with surgery and adjuvant RT and may also include CT or hormonal therapy. Advanced-stage BC focuses on controlling the disease and managing symptoms, with treatment options including CT, targeted therapy, hormonal therapy, or a combination (96, 97). The level of care provided for breast cancer is constantly changing, with emerging therapies and approaches, including immunotherapy, being researched, and evaluated through clinical trials. Likewise, research focusing on the immune landscape is underway to establish immune-based predictive biomarkers to improve patient stratification (98). As RT is primarily administered as an adjuvant treatment following surgery (96), efforts to identify biomarkers are shifting their focus toward the systemic immune landscape, rather than the iTME.

### Systemic immune landscape of BC and radiotherapy

Radiation therapy is often associated with lymphopenia in breast cancer patients since the lung and heart, the two organs that contain blood in the thorax, are situated within the radiation field. This systemic state seems to be persistent even one year after RT (99). Therefore, it is crucial for clinicians to create dependable dosimetric models for use as a benchmark in dose prescription and treatment planning. Chen et al. investigated the connection between effective dose to the circulating immune cells (EDIC) and radiation-induced lymphopenia (RIL) in a group of breast cancer patients (100). The EDIC model calculates the dose based on the portion of blood flow to the lung, heart, and liver, as well as the body surface area exposed to radiation (101). As the EDIC value increased, the RIL rose correspondingly, suggesting that this model accurately reflects the dosimetric factor that directly affects lymphopenia. A more thorough examination of the subpopulations impacted by adjuvant RT revealed a decrease in T-cells and platelets, but not immunosuppressive myeloid subpopulations (CD13+CD56+ cells) (102). This comes into contradiction with the meta-analysis by Wang et al., which shows no significant difference in peripheral blood T cells after RT (18). However, it is important to highlight that the time point of the blood sampling after RT (immediately after or 48h later, etc) plays a role in the result recorded. A study looking at the T cell compartment in the blood found that adjuvant RT increased the memory and regulatory CD4+ T cells (103) which agrees with the increase in T helper cells during RT seen by Sage et al. (99). It is yet to be determined if these T helper cells are a representation of Tregs, and whether alternative therapeutic options could be employed to prevent immunosuppression. Given these discoveries, it is essential to conduct further research on peripheral blood immune populations to establish dependable biomarkers for monitoring disease progression and potential treatment combinations.

### iTME of BC and radiotherapy in human samples and pre-clinical models

The reciprocal relationship between RT and iTME has not been extensively studied in breast cancer, as RT is not typically used as a NAT treatment for this type of cancer (104). However, the specific immune cells present in the iTME appear to be important for disease progression, as demonstrated by Schnellhardt et al. (105), who found that high densities of B and memory cells were associated with reduced DFS in early-stage BC. This led to the development of a prognostic score based on the cell densities of these subpopulations in the tumor core and stroma to determine different patient risk groups. Although patient data is limited, pre-clinical data can provide insight into the relationship between RT and iTME in breast cancer. *In vitro* studies have shown that RT of breast cancer cell lines (2 Gy or 5 Gy) resulted in an upregulation of the immune checkpoint molecules PD-L1 and PD-L2, which may have implications for combination with immunotherapy (106). The study of iTME in breast cancer aims to establish treatment combinations rather than biomarkers, as RT is mainly given in an adjuvant setting and thus the iTME is unlikely to affect treatment response.

Thus, radiotherapy is a frequently used supplementary treatment for breast cancer and has the potential to affect the immune system. The occurrence of low lymphocyte count induced by radiotherapy can contribute to the creation of immune tolerance. Additionally, radiotherapy may alter the composition of different subsets of the body’s immune system, affecting T cells while having no impact on myeloid suppressor cells. To better understand these changes and develop more effective radiotherapy regimens with or without concordant immunotherapy, further research is necessary.

### Cervical cancer

Cervical cancer ranks second in incidence and mortality among women from countries with Human Development Index (HDI) (107) following breast cancer and is developed following an HPV viral infection (108). Research in the tumor microenvironment shows a highly heterogeneous profile that can be altered with the use of radiation.

#### Systemic immune landscape of cervical cancer and radiotherapy

Comparing the dynamic changes in the systemic immune contexture with the landscape of the TME can be very interesting to further understand the interactions taking place. In this scope, a retrospective study compared the effect of CRT in cervical cancer patients using blood samples and cervical brushing specimens at the same time points. CRT seemed to have a stronger effect on the tumor microenvironment since there was a significant decrease of the T helper cells intratumorally that was not seen in the periphery and more interestingly there was an increase in the activated T cells (CD69+ cells) only in the cervix (109).

#### iTME of cervical cancer and radiotherapy

Radiotherapy can have stimulatory effects on the iTME as was seen in a prospective analysis of tumor-associated macrophages (TAMs) of patients treated with radical RT (110). A RT-dependent increase in the number of TAMs in cervical cancer tissue and a parallel shift towards the M1-like or pro-tumoral phenotypic state of macrophages (increased expression of CCR7 and decreased expression of CD163) was observed. Extracellular vesicles (EVs) were found to be responsible for the reprogramming of TAMs and the increased phagocytic activity ex vivo, although further pre-clinical investigation is needed.

As far as lymphocytes are concerned, Li et al. (111). prospectively analyzed the dynamic changes in the iTME of patients with cervical cancer that were treated with CRT. The number of CD4+ and CD8+ T cells in the tumor decreased at the same time as PD-1 expression and TCR diversity declined. In accordance with these results, another study also showed significant reduced cytotoxic (CD8+) T cell and T regulatory cell infiltration after RT as seen in paired pre-RT biopsies and post-RT surgical specimens (112). Interestingly, they could not see a difference in the effect of RT on the PD-1 and PD-L1 expressing cells. Most notable was the data from Mori et al. (113), where they saw that the stromal CD8+ T cells increased only in the patients receiving RT alone while the combination of CRT caused a reduction in the same population. Although in contradiction with previous data that observed an increase in CD3+ T cells when using chemotherapy, this might suggest a systemic effect of chemotherapy that is mirrored in the iTME (114).

The data gathered from studies of cervical cancer suggest a highly heterogeneous environment, but there is a trend in which RT has an ablative effect on intratumorally T cells (115). This needs to be considered when the question of the implementation of immunotherapy arises since the cold iTME may present a greater risk of toxicity.

### Ovarian cancer

Ovarian cancer is the most lethal female reproductive malignancy representing 1% of all new cancer cases and it is often characterized by late-stage diagnosis (116). Therapy usually consists of debulking surgery with neoadjuvant or adjuvant chemotherapy to reduce the tumor burden (117). Neoadjuvant chemotherapy has been shown to increase the infiltration of T regulatory cells (118) and stromal lymphocytes (119) which has implications for the use of immunotherapy. In recent years, new immunotherapeutic approaches such as ICIs, chimeric antigen receptor (CAR)- and TCR-engineered T cells are used therefore the immune landscape is brought to the forefront of ovarian cancer research (120). RT is rarely used in ovarian cancer, as these tumors spread through the peritoneal cavity, conventional radiation therapy targeting large volumes being too risky because of toxicity (121). To bypass this large-volume toxicity, researchers used low-dose radiation therapy (LDRT) in an *in vivo* orthotopic ovarian cancer model to reprogram the TME and enable immunotherapy to work more effectively (122). They observed an IFNγ-depended intra-tumoral influx of cytotoxic T cells, T helper cells and monocytes following low-dose radiation of 1Gy that can be combined with immunotherapy for a synergistic effect toward tumor regression. Most intriguing were the results of the following clinical study on patients with cold tumors where they also showcased an increase in T helper cells after radiation with subsequent tumor responsiveness to therapy. It would be interesting to see in the future more studies in larger cohorts to examine the combination of low-dose radiation therapy and immunotherapy in ovarian cancer.

### Prostate cancer

Prostate cancer is the second most common cancer type in men above 50 years old (123). The biomarker that is used throughout prostate cancer follow-up is the prostate-specific antigen (PSA), and in recent years immunological parameters have been investigated for their prognostic and predictive validity (124).

#### Systemic immune landscape of prostate cancer and radiotherapy

Normofractionated RT was found to temporally decrease the density of T and B cells in a prospective immuno-modulating study (125). Moreover, the peripheral subsets of regulatory T cells and NK cells increased during treatment, which is in line with pre-clinical prostate cancer models (126, 127). Radiotherapy with charged particles such as carbon ions (CIR or carbon ion radiotherapy) was used in a cohort of prostate cancer patients to ensure better dose distribution and greater relative biological effectiveness (RBE). Interestingly, among the immunomodulatory effects of CIR, they found a persistent increase in T helper cells during follow-up in parallel with an increase in CD19+ cells associated with a humoral activity. In addition, after CIR, the ratio of T helper to cytotoxic T cells (CD4+/CD8+) was higher in responders than in non-responders, indicating the immunological status to predict CIR outcome (128). Since CIR therapy has not been studied extensively yet, more research is needed to determine its effect on the immune landscape.

Photon radiotherapy can have an ablative effect, which can be avoided by using particle radiotherapy, such as CIR, however since there is further investigation is needed.

#### iTME of prostate cancer and radiotherapy

According to one of the first studies that looked at the effects of prostate SBRT on the immune landscape, radiation increases CD68 and CD163 macrophages while harming CD8+ T cells (126). The authors suggest further investigation with transcriptomic analysis in order to connect these alterations in the iTME with intratumoral cytokine profile. Moreover, macrophages showcase vast and complex plasticity in cases where they express mixed M1 and M2 surface markers (129). Therefore, the in depth transcriptomic analysis of the myeloid subpopulations will shed a light on their role in the iTME of prostate patients after RT.

#### iTME of prostate cancer and radiotherapy in a pre-clinical model

After SBRT, the iTME shifts towards a more immunosuppressive phenotype as evidenced by the increase in M2 macrophages and the decrease in cytotoxic T cells. An *in vivo* murine prostate cancer tumor model, presented after irradiation, an increase in Treg populations in the spleen and other organs (127). In particular, when TRAMP-C1 tumors were locally irradiated, this resulted in a greater percentage of CD4+CD25^hi^Foxp3+ cells in the spleen while at the same time, all these cells expressed the exonuclease CD39. As a result, it appears that Tregs are not only escaping the harmful effects of this specific radiation dose regimen but are also retaining their immunosuppressive capacity.

Altogether, we can conclude that RT in prostate cancer is driving the TME toward immunosuppression. In one study by Fang Yu et al. (130), they tried to render the ‘‘cold’’ tumors ‘‘hot’’ by local injections of interleukin-12 in combination with RT to boost the immune system. They saw recruitment of Th1 and CD8+ T cells in the tumors after the combination therapy which resulted in a significant decrease in tumor size compared to the control group with RT alone. It would be interesting to investigate the effects on immune-checkpoint molecule expression in order to evaluate whether immunotherapy could be beneficial for these patients.

### Pancreatic cancer

The most prevalent form of this cancer is the exocrine pancreatic ductal adenocarcinoma (PDAC) (131), which is among the most aggressive solid tumors due to the highly immunosuppressive TME and poor response to chemotherapy, radiotherapy and immunotherapy. This poor treatment response may in part be explained by the dense desmoplastic stroma and the abundance of immunosuppressive cells in the PDAC TME, which excludes antitumoral T cells, resulting in a *cold* tumor (132, 133). Radiation can, therefore, be beneficial in boosting the immune system’s response to systemic therapies and in achieving tumor regression by altering the TME (134, 135).

#### Systemic immune landscape of pancreatic cancer and radiotherapy

In the context of pancreatic cancer, prognostic and predictive value may be conferred by systemic inflammatory markers. The survival rate of patients with locally advanced PDAC treated with SBRT was lower when the neutrophil-to-lymphocyte ratio (NLR) was high before treatment (136). Moreover, when localized pancreatic cancer is treated with anti-PD-1 antibodies, NLR is elevated due to lymphocyte depletion after SBRT and associated with worse survival (137). Low lymphocyte-to-monocyte ratio (LMR) after nCRT therapy followed by surgery was a poor predictor for prognosis in patients with borderline resectable pancreatic cancer (BRPC) (138). The poorer survival rate in BRPC and locally advanced unresectable pancreatic cancer (LAUPC) was confirmed to be associated with a high monocyte count but also a low γδ T cell count (139). γδ T cells are lymphocytes that are found in great numbers in the intestine and dermis (140). As this *unconventional* lymphocyte subset requires no cross-presentation of MHC, it has demonstrated enhanced effector capacity *in vitro*, which can explain why low numbers of it are associated with poor survival in humans (141). In addition, in a randomized controlled trial, serum levels of the pro-tumor CXCL8 cytokine were associated with a favorable prognosis in patients undergoing CRT for pancreatic cancer (37). As a result of RT-induced release of CXCL8 from tumor cells, NK infiltrates increased in PDAC tumors with cytotoxic gene signatures. It appears that CXCL8 plays a role in activating immune surveillance against tumors after RT, however, a detailed analysis of the systemic cytokine profile of patients is needed to draw more definitive conclusions. Additionally, since the NLR and LMR ratios have gained prominence as prognostic markers for pancreatic cancer (142, 143), multicenter studies with larger cohorts are recommended to implement these markers in regular clinical practice.

#### iTME of PDAC and radiotherapy in human samples

Radiation of the TME can induce immunogenic cell death since the increased antigen presentation stimulates an immune response against tumor cells. Hence, after nCRT an increased expression of DAMPs such as calreticulin, Hsp70, and MICA/B is observed. Moreover, there is an increase in the absolute number of T helper and cytotoxic cells (CD4+ and CD8+ respectively) and most importantly the Treg/TIL ratio is decreased and can be used as a predictor for longer survival (144).

Tumor samples that were treated with SBRT presented with increased immunogenic cell death (ICD) and PD-1+ T effector infiltrate compared with the untreated control group (145). A spatial analysis of this subpopulation revealed that these cells to be were outnumbered by surrounding immunosuppressive myeloid populations (monocytes, macrophages, and granulocytes), which could limit their function. Although the writers acknowledge that these cells are prone to exhaustion, they refer to the subpopulation of cytotoxic T cells expressing PD-1 as activated T cells to further support their claim that combining ICI therapy with RT will improve therapeutic outcomes in PDAC.

A detailed analysis of how radiation affects the immune landscape will shed light on the possibility of using CRT to downgrade PDAD tumors in a neoadjuvant setting (146, 147). In a study comparing nCRT to upfront surgery the number of T helper cells, B cells and Tregs decreased in the stroma but not in the tumor core (38). In addition, only M2-like macrophages in the tumor core were a reliable predictor of early disease recurrence after nCRT for PDAC. An interesting finding was that M2 TAM infiltration (CD206+ cells) decreased more in female PDAC patients after nCRT (148). The immune landscape and RT response in patients with PDAC may thus be affected by biological sex; however, conclusions cannot be drawn until further research on the subject is conducted.

#### iTME of PDAC and radiotherapy in pre-clinical models

With a murine pancreatic cancer model, Ye et al. evaluated the ability of SBRT to induce immunogenic cell death (149). They found SBRT with concurrent chemotherapy to increase antigen presentation and cytotoxic T-cell infiltration. The infiltrated cytotoxic T cells had an increased capacity for secreting IFNγ and elucidating an immune reaction. In another study, low-dose irradiation of pancreatic tumors in mice resulted in increased numbers of iNOS pro-inflammatory macrophages and subsequently the recruitment of T cells into the tumors (150). However, the data are conflicting since in an orthotopic pancreatic murine tumor model the proportion of M2 anti-inflammantory macrophages increased upon irradiation (151). Moreover, the shift towards an immunosuppressive milieu was further backed up by data showing fewer CD8 T cells and more T-helper 2 and T-regulatory cells present in the irradiated pancreata compared to controls. An additional study found that after low-dose irradiation of insulinomas, iNOS was upregulated in the peritoneal macrophages, whereas markers of M2 macrophages were downregulated, suggesting a skewing towards M1 macrophages after RT (152). Thus, in this subtype of pancreatic cancer, RT seems to be beneficial since it shifts the balance towards anti-tumoral effects. Further studies need to be performed to give a definite result.

In conclusion, PDAC TME is a complex network of interactions with different immune cell populations that can confer predictive validity as biomarkers. Consequently, some patients may benefit more from a combination of radiation and immunotherapy targeting these cells in order to achieve the best results.

### Colorectal cancer

Colorectal cancer (CRC) is the third most common cancer type worldwide (153) and, due to lifestyle changes, it is becoming more common among individuals younger than 50 years old (154). Cancers of the colon (sigmoid, descending, transverse, and ascending) and rectum, which make up parts of the large intestine, are classified as colorectal cancers (155).

### Colon cancer

#### iTME of colon cancer and radiotherapy in pre-clinical models and human samples

Radiation was found to increase the infiltration of immune cells into colon tumors in a murine model (156). More specifically, the number of macrophages (CD11b^high^/F4-80+) increased on day 5 after hypofractionated radiotherapy while the number of APCs (MHCII +) and cytotoxic T cells increased significantly on day 8 compared to the non-irradiated controls. It is interesting to note that the infiltration of these cells only takes place during a very short period something that needs to be considered when designing treatment schedules. The importance of timing was also highlighted by Gerber et al. (157). Since they could distinguish the responsive tumors from the non-responsive ones as early as 4 days after irradiation. Most notably, in the tumors responding well, they could see an increase in the levels of IFN-γ and the infiltration of immune cells was increased to further boost the activity of the cytotoxic T cells. In a syngeneic colon cancer model, Joseph et al. (158) observed an expansion of CD8+ T cells after CRT and more specifically of tumor-specific CD3+ tissue-resident memory cells (T_RM_). Dissecting the molecular mechanism behind this expansion and activation, the researchers identified the tumor-draining lymph node (TDLN) resident CD103+ dendritic cells to be the drivers of this priming (158). Based on other preclinical data, radiation enhances antigen-presenting cells’ activity, as expected due to immunogenic cell death (ICD). A therapy targeting these cells can develop in the form of antibodies against checkpoint molecules such as CD47 (159, 160) that may synergize with radiation to promote tumor regression.

In human CRC biopsy samples, Schaue et al. focused on the effect of radiation on tumor-specific T-cell reactivity (161). After completing CRT, they found that tumor-specific T cells increased in the majority of patients. Moreover, the T cells expressing survivin, a tumor-specific antigen found in many cancers and believed to be immunogenic (162), did not decrease indicating that the treatment did not impair their ability to respond.

### Rectal cancer

Although, rectal cancer (RC) and colon cancer are commonly lumped together under the umbrella term colorectal cancer, but in recent years, there has been growing evidence that they yield more differences than just the anatomical location. In particular, they differ in embryonic origin, physiological function, anatomy, metastatic patterns, and first-line therapy, so it is important to separate the two when studying them (163). Rectal adenocarcinomas, the most prevalent form of rectal cancer, develop from malignant epithelial cells in the last 15cm of the colon, and for patients presenting with locally advanced rectal adenocarcinoma (LARC) neoadjuvant treatment that includes radiation is commonly prescribed (164). At the same time, total mesorectal excision surgery, which removes the entire rectum and the surrounding mesorectum with the pararectal lymph nodes, is the standard of care after neoadjuvant treatment, and negatively impacts the quality of life (QoL) of the patient (165). There is increasing interest in non-surgical strategies to decrease treatment-related toxicity after complete tumor remission due to neoadjuvant treatment (166). Therefore, patients that present with early-disease stages, responding better to nCRT, could be spared of the detrimental effects on the QoL of total mesorectal excision if they could be accurately stratified before surgery. As a result, patients could be categorized for a wait-and-watch approach to spare the organ and eventually achieve local control based on the immune landscape, both systemically and intratumorally.

#### Systemic immune landscape of rectal cancer and radiotherapy

The neutrophil, platelet, and lymphocyte count after CRT have been associated with prognosis in multiple cancer types. In rectal cancer, a high systemic inflammation index (SII) was associated with poor OS after CRT in patients with rectal cancer (167). The SII is a measure of the neutrophil to platelet count with the total lymphocytes and can accurately describe the systemic immune landscape (168).

#### iTME of rectal cancer and radiotherapy in human samples

Recently, immunological tissue-based biomarkers started to gain momentum with the most noticeable being the Immunoscore which measures the density of CD3+ and CD8+ TILs (169). To date, the Immunoscore has been confirmed to have a prognostic-only value after a meta-analysis of 10.000 colon cancer patients showing a high immunoscore (IS=4) to be correlated with the lowest risk of recurrence, the longest OS and the longest DFS (35, 170). As far as radiation is concerned, a biopsy-based immunoscore (ISb) was successfully used to predict response to neoadjuvant chemotherapy and selection for watch-and-wait therapy in patients with LARC (32). Moreover, the immunoscore was analyzed in pairs of biopsies and surgical samples of rectal cancer patients that received nCRT. After CRT Anitei et al. saw a significant increase in the infiltrating CD3+ and CD8+ cells that also correlated with tumor downstaging marking the immunoscore as a good potential biomarker for response to RT (36). Furthermore, an increase in the influx of CD3+ and CD8+ TILs was observed in post-treatment samples compared to the pre-treated counterparts suggesting an immunogenic effect of radiation (171). The most recent validation for immunoscore as a strong predictive biomarker comes from a study by Sinicrope et al. (172), where the higher DFS of patients with stage III colorectal cancer was predicted by their high immunoscore using Immunoscore^®^ Colon CE-IVD test standardized assay indented for routine clinical practice (173). Given all of these, there is a strong correlation between immunoscore and response to treatment. However, functional evaluation of T cells is always required since high density does not necessarily indicates cytotoxic activity.

Another study by Mirjolet et al. analyzed the immune infiltration of cytotoxic T cells and Tregs in biopsies and surgical sample pairs of patients that received preoperative RT for LARC. The infiltration was assessed by calculation of the CD8+/FoxP3+ ratio in the epithelium and stromal compartment. A decrease in Treg populations was observed after the use of RT whereas the density of cytotoxic T cells remained unchanged leading to an overall increase in the ratio (174). To better support these data, another study also found an increase in the cytotoxic T-cell density in samples taken during RT compared to the pre-treatment samples (175). As observed in patients by Joseph et al. (158), CRT also polarizes the iTME towards an activated and memory Th1 transcriptomic signature. Interestingly, the same group saw a higher expression of PD-L1 by immune cells in CRT compared to RT alone, which could be exploited later with the use of ICI. The presence of T helper cells in the TME influences other cell populations such as cytotoxic T cells as was shown by mIHC of the different cell neighborhoods (46).

Following these data, Kamran et al. (34) evaluated transcriptomics data from pre- and post-CRT–matched tumor samples from a cohort of rectal cancer patients that included several non-responders (NR). As expected, CRT changed the immunological profile with an increase in the immune cell infiltration of naïve B cells, cytotoxic T cells, monocytes, pro-tumoral macrophages and resting mast cells. On the other hand, CRT seemed to negatively affect the memory B cells and activated mast cells that were abundant in the pre-CRT samples.

#### iTME of rectal cancer and radiotherapy in pre-clinical models

Irradiating *ex-vivo* non-treated human rectal cancer tissue and assessing phenotypically the macrophage populations with flow cytometry, Stary et al., observed polarization of the irradiated TAMs towards an M1-like phenotype which was functionally supported by *in vitro* data showing increased levels of phagocytosis after low-dose radiation. More interestingly, they observed an increase in the M1/M2 ratio in rectal cancer patients that underwent hyper-fractionated short-course RT compared to treatment-naïve specimens from patients of the same clinical TNM stage (176). In another study, Wilkins et al. analyzed the immune gene expression profiles (GEP) of sample pairs of pre-treatment biopsy and surgical excision after RT/CRT and there was an increase of the M2-like phenotypic marker CD163 in non-responders to RT. In addition, after RT, the good responders adopted an immune-hot phenotype with increased T-cell infiltration, upregulation of inflammatory pathways and ‘‘wound repair’’ stroma phenotype (33).

**Figure 2:**
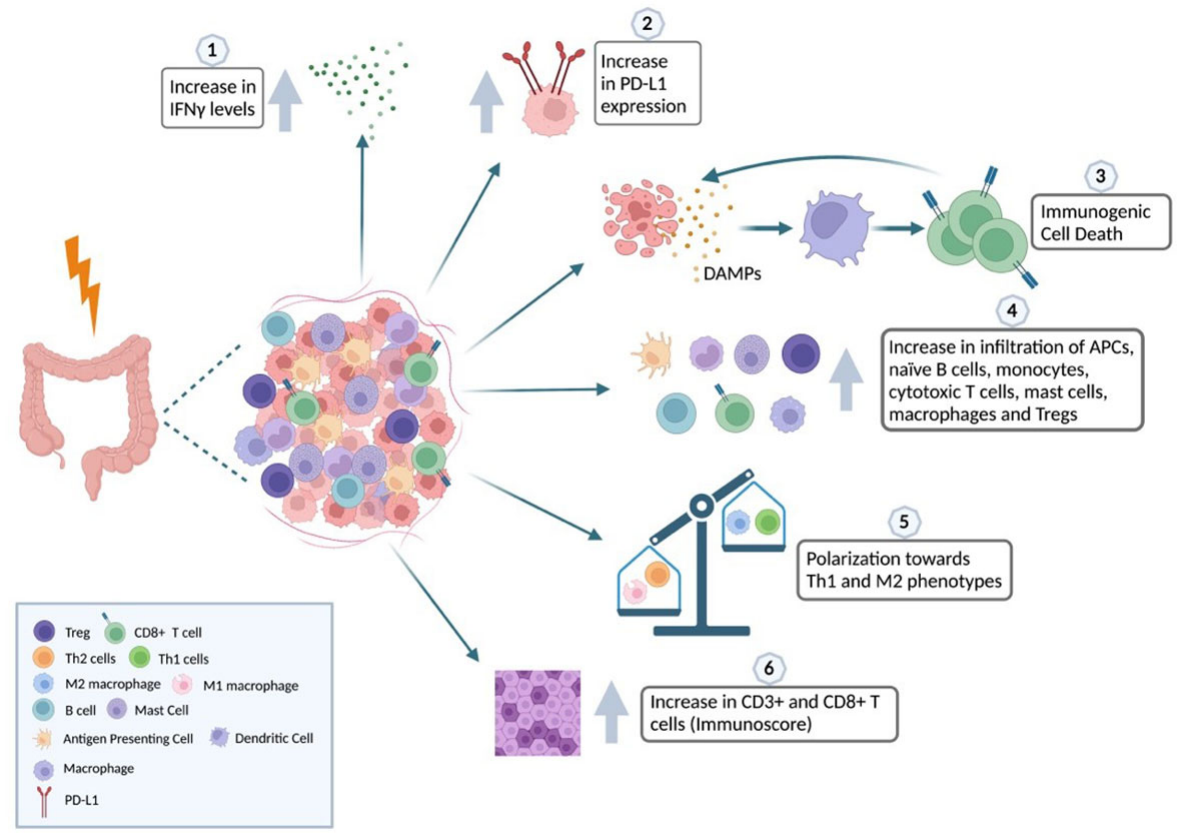
Impact of radiation in the iTME of colorectal cancer. The relationship between radiotherapy and the immune landscape of CRC is complex and context-dependent. Radiotherapy has the potential to harm normal tissues and stimulate the secretion of inflammatory cytokines like IFNγ (1). This, in turn, can recruit immune cells that hinder the immune response against the tumor. Additionally, radiotherapy can trigger the upregulation of immune checkpoint molecules that block immune cell activity (2). However, radiotherapy can also induce immunogenic cell death (ICD), a process where cancer cells release molecules that activate the immune system and promote an immune response against the tumor (3). ICD can increase the infiltration of immune cells, such as APCs, naïve B cells, monocytes, cytotoxic T cells, mast cells, macrophages, and Tregs, into the tumor microenvironment (4). Furthermore, radiotherapy can shift the balance of macrophages towards a pro-tumoral phenotype and T-helper cells towards the Th1 phenotype involved in cell-mediated immunity (5). Effective use of radiotherapy in CRC treatment depends on various factors, such as tumor stage and location, patient characteristics, and the timing and sequencing of radiotherapy with other treatments, including chemotherapy and immunotherapy. Using biomarkers, such as the Immunoscore based on the densities of CD3+ and CD8+ T cells, can help classify patients better, as it has been shown to increase after RT and correlates with tumor downstaging and potential response to therapy (6). Created with BioRender.com.

It is challenging to study the immune landscape associated with gastrointestinal cancer because of its heterogeneous nature (see **Figure 2**) but we can observe a shift towards M2 polarization after RT and an increase in the influx of CD8+ T cells intratumorally in the same way as in other tumor types.

### Anal cancer

Anal squamous cell carcinoma (ASCC) is a rare disease whose incidence is increasing in the western world (177) and is linked to human papillomavirus (HPV) (178). In a cohort of locally advanced anal carcinoma patients with HPV-positive and negative cases, the majority were treated with CRT or RT alone. After a follow-up period of 20 years CD3+, CD8+ and PD-1+ tumor-infiltrating lymphocytes were revealed as favorable prognostic markers after radiation (179). As we have seen in other types of cancer, this result is not uncommon. Worth noting, that CD4+ T cell numbers in either too low or too high density, which is considered a hormetic effect (180), also predicted more favorable outcomes for the patients. The authors hypothesized that the balance between the different subpopulations of CD4+ T cells (Th1, Th2, Th17, Treg) is the main reason why good prognosis can be associated with both low and high concentrations of T helper cells. As pro-inflammatory and pro-tumor immune cells share common markers, phenotypic characterization of these subpopulations is valuable for the understanding of TME. There is a need for further investigation of this phenomenon, but anal cancer immune landscape studies are limited following RT. Luckily, the pattern of favorable prognosis correlated with hot TME can be seen (and thus studied) in other cancers as well.

### Liver cancer

#### Hepatocellular carcinoma

Hepatocellular carcinoma (HCC) is the most common form of liver cancer accounting for more than 80% of the cases and has a higher mortality rate. There is great heterogeneity in HCC which can be expanded into three different levels: interpatient heterogeneity where the differences can be seen from patient to patient, intertumoral heterogeneity from one tumor nodule to another and intratumoral heterogeneity between the different regions of the same tumor (181, 182). This vast heterogeneity can be seen in the iTME and influences the response to the various treatments (183). Craciun et al. showed that after selective internal radiation therapy (SIRT) the infiltration of T helper and cytotoxic, and granzyme B expression was significantly increased in patients with HCC indicating a shift towards an immunostimulatory milieu (184).

Systemically, lymphopenia is a common side effect of radiation in the immune landscape and can be used as a biomarker. Also in HCC, the NLR is elevated after SIRT. An elevated NLR or low lymphocyte count is a poor prognosis factor for disease progression and should be considered for decision-making during and after treatment (31). A short fractionation regimen of RT could potentially spare the lymphocyte population from depleting and speed the recovery of the patient after therapy (185). This result was confirmed by another cohort of HCC where the elevated number of circulating lymphoid cell populations was correlated with better OS after irradiation (33).

Finally, to unravel the abscopal effect, the indirect shrinking of the tumors outside the irradiated field due to systemic immune modulatory properties of the radiotherapy, Hee Park et al. (186) used a murine model of HCC which revealed an increase in the number of tumor-specific T cells and IFN expression in splenocytes after RT. Moreover, a subsequent increase of dendritic cells in the tumor-draining lymph nodes and of cytotoxic T cells in the metastatic tumor site further supports the hypothesis of fimmu.2023.1148692the irradiation-induced activation of the immune system (187).

#### Intrahepatic cholangiocarcinoma

Primary liver cancer has two common subtypes: HCC developing from hepatocytes and intrahepatic cholangiocarcinoma (ICC) that arise from the small intrahepatic bile duct epithelium (188).

When looking at patients with ICC after hypofractionated proton therapy, strangely a longer OS was significantly correlated with a higher number of naïve (CD4+CD25+) and memory (CD4+CD127+) T cells in the blood at the beginning of hypofractionated proton therapy (189). For HCC patients, the same subpopulations of cells did not have any significance, since only the activated cytotoxic T cells (CD8+CD25+) mid-treatment were a strong prognostic factor for survival. In another study, the MDSC monocytic subtype of CD14^+^HLA-DR^−/low^ which is part of the immature myeloid cell lineage and is thought to be highly immunosuppressive significantly increased in the blood of HCC patients after radiation therapy (3D-CRT or IMRT) (190).

Further studies are required to clarify the effects of radiation on the immune landscape based on the limited results from the two subtypes of liver cancer. The radiation therapy for HCC, however, has the potential to increase the infiltration of TIL, as it does for other cancer types, which may be beneficial to patients.

### Skin cancer

Skin cancer includes basal cell carcinoma (BCC) and squamous cell carcinoma (SCC), together with merkel cell cancer collectively named non-melanoma skin cancers (NMSC) and melanoma. A skin malignancy’s type and stage at the time of diagnosis determines the treatment options. In terms of cosmetic and functional outcomes, radiotherapy may offer better tissue preservation in definitive, adjuvant and palliative settings than surgery. NMSCs are radioresponsive tumors and have local control rates of 90-95% after RT (191). Melanoma is less radiosensitive but interestingly immunotherapy is particularly effective and the combination with RT is believed to yield promising results (192, 193). It is possible to identify potential markers to guide future treatment plans by analyzing the immune landscape after RT for skin cancer (194). In this scope, Bazyar et al. (195) assessed the immunological changes using micro planar radiation therapy (MRT), a technique that spatially delivers high-dose beams (peaks) in certain tumor regions sparing other areas (valleys). The advantage of this technique is believed to be the greater protective effect on normal tissue and tumoral specificity. In their study, micro planar radiation therapy was compared to CRT in a radioresistant B16-F10 murine melanoma model yielding better tumor regression and survival. Most importantly, this effect was attributed to a higher influx of CD8+ T cells and a lower influx of intratumoral Tregs.

## Perspectives

The heterogeneity of primary tumors among patients (interpatient heterogeneity) and even within the same patient (inter– and intra- tumoral heterogeneity) hinders oncology. It is imperative to overcome the bottleneck of heterogeneity along with a shift towards personalized treatment so medical decisions and interventions can be tailored to the individual. The study and elucidation of the iTME can help in both directions since on the one hand, the immune contexture can be common between different histological cancer types as analyzed in this review, and on the other hand, the immunological profile of the tumor can serve as a guide to treatment.

Several advantages of radiotherapy are offered to patients, such as tumor downstaging for better growth control and easier surgical removal, localized treatment with less toxicity in healthy organs than systemic chemotherapy and ICIs, as well as the recent option of organ preservation, which increases the quality of life for patients. Enhancing radiotherapy’s effectiveness is therefore in the patient’s best interests. Various factors can affect the response to radiotherapy, one of which is the immune landscape systemically and/or intratumorally. A wide range of factors influences the effect of radiotherapy on the immune landscape, including dose and fractionation, target volume, radiation fields, and histological type and grade. It is important to understand how radiotherapy changes the immune landscape, as well as how the existing immune landscape influences the patient’s response, in order to develop therapeutic interventions that will improve the efficacy of radiotherapy and convert some ineffective responses into effective ones.

## Author contributions

CI collected the data and drafted the manuscript. MK revised the manuscript. DG, PM, LV and CB provided additional revisions. All authors contributed to the article and approved the submitted version.

## Glossary

**Table:**

AAC	Anal Adenocarcinoma
APCs	Antigen Presenting Cells
ASCC	Anal Cell Carcinoma
BCC	Basal Cell Carcinoma
BC	Breast Cancer
CAFs	Cancer Associated Fibroblasts
CAR	Chimeric Antigen Receptor
CCRT	Concurrent Chemoradiotherapy
CIR	Carbon Ion Radiotherapy
CMS	Consensus Molecular Subtypes
CRC	Colorectal Cancer
CRT	Chemoradiotherapy
CTLA-4	Cytotoxic T-Lymphocyte Associated Protein 4
CTV	Clinical Target Volume
DAMPs	Damage-Associated Molecular Patterns
DFS	Disease Free Survival
EC	Esophageal Cancer
FC	Flow Cytometry
FFPE	Formalin Fixed Paraffin Embedded
HCC	Hepatocellular Carcinoma
HNSCC	Head and Neck Squamous Cell Carcinoma
HPV	Human Papillomavirus
ICC	Intrahepatic Cholangiocarcinoma
ICI	Immune Checkpoint Inhibitor
IDO	Indoleamine 2,3-dioxygenase
IE/F	Immune/Fibrotic TME Subtypes
IFN-γ	Interferon gamma
IHC	Immunohistochemistry
IM	Invasive Margin
IMRT	Intensity - Modulated Radiotherapy
IS	Immunoscore
ITCs	Intratumorly T cells Clonotypes
iTME	Immune Tumor Microenvironment
LARC	Locally Advanced Rectal Cancer
LMR	Lymphocyte-to-Monocyte Ratio
LS-SCLC	Limited Stage Small Cell Lung Cancer
LUAD	Lung Adenocarcinoma
MDSCs	Myeloid-Derived Suppressor Cells
MHC	Major Histocompatibility Complex
mIHC	Multiplex Immunohistochemistry
nCRT	Neoadjuvant Chemoradiotherapy
NK	Natural Killer Cells
NLR	Neutrophil-to-Lymphocyte Ratio
NPC	Nasopharyngeal Carcinoma
NSCLC	Non Small Cell Lung Cancer
OAC	Esophageal Adenocarcinoma
OS	Overall Survival
PBMCs	Peripheral Blood Mononuclear Cells
PD-L1	Programmed Death Ligand
PDAC	Pancreatic Ductal Adenocarcinoma
PFS	Progression Free Survival
PSA	Prostate Specific Antigen
QoL	Quality of Life
RT	Radiotherapy
SBRT	Stereotactic Body Radiotherapy
SCC	Squamous Cell Carcinoma
TAMs	Tumor Associated Macrophages
TANs	Tumor Associated Neutrophils
TCR	T Cell Receptor
TDLN	Tumor Draining Lymph Node
TGFβ	Tumor Growth Factor β
Th	T helper cells
TILs	Tumor Infiltrating Lymphocytes
TMB	Mutational Burden
TME	Tumor Microenvironment
TNM	TNM Classification of Malignant Tumors
RT	Radiotherapy
Tregs	Regulatory T cells
TS	Tumor Core
